# Implementing IPE in a Workplace Setting: Educational Design Research Promotes Transformative Participation

**DOI:** 10.5334/pme.1546

**Published:** 2025-01-23

**Authors:** Marco A. C. Versluis, Lizzy-Sara Zöllner, Sofia Papadopoulou, Relinde van der Stouwe, Marie-Josée C. de Haan-Gremme, Anna H. C. Tsiamparlis-Wildeboer, Héleen Helmholt, Marco Antonio de Carvalho-Filho

**Affiliations:** 1 Department of Obstetrics and Gynaecology, University Medical Center Groningen, The Netherlands; 2 Wenckebach Institute, Lifelong Learning, Education and Assessment Research Network (LEARN), University of Groningen, University Medical Centre Groningen, Groningen, The Netherlands; 3 Midwifery Academy Amsterdam Groningen, InHolland, Groningen, The Netherlands; 4 University of Groningen, University Medical Center Groningen, Department of Primary and Long-term Care UMCG, Groningen, The Netherlands; 5 Amsterdam UMC, location Vrije Universiteit Amsterdam, Midwifery Science, De Boelelaan 1117, Amsterdam, The Netherlands; 6 Amsterdam Public Health, Quality of Care, Amsterdam, The Netherlands

## Abstract

**Background:**

Educators struggle to implement Interprofessional Education (IPE) in workplace settings. We adopted an educational design research (EDR) approach to implement an IPE activity and establish design principles supporting IPE implementation in workplace settings.

**Method:**

We adopted an iterative process of analysis/exploration, design/construction and evaluation/reflection. We performed a scoping review, visited examples of IPE initiatives and involved workplace professionals to define preliminary design principles for implementation. An IPE activity was implemented where students from nursing, midwifery and medicine care for patients together. Continuous reflection during the EDR process supported the refinement of design principles.

**Results:**

We describe 14 design principles for implementation of IPE: (1) Set an objective; (2) Make the project evidence informed and theory driven; (3) Nurture a growth mindset; (4) Stimulate transformative participation; (5) Be aware of culture; (6) Support faculty members; (7) Align learning outcomes (8) Design formative and reflective assessment methods; (9) Position within an authentic context; (10) Facilitate informal interaction; (11) Balance patients’ safety with attributing responsibility; (12) Align with the workplace, seize opportunities to improve interprofessional collaboration; (13) Evaluate the implementation; AND (14) Trust the process. The design principles related to three overarching concerns describing IPE implementation as a change process: patient safety, workflow and culture.

**Discussion:**

The 14 design principles support context sensitive IPE implementation in the workplace. The EDR approach nurtured transformative participation, empowering stakeholders to participate and contribute to design and decision making. This resulted in an evidence informed, interprofessional cocreation process *in* and *with* the workplace that was aligned with existing workflow and organizational culture.

## Introduction

IPE occurs when students from two or more professions learn about, from, and with each other to enable effective collaboration and improve health outcomes [1,2,3,4,5,6,7]. It is a celebrated approach to healthcare education and plays an important role in addressing the current human resource crisis that threatens healthcare systems worldwide [8,9]. IPE holds a promise not only to break the barriers between professional silos, but also to adapt to changes in healthcare demands posed by societal developments [8,9,10]. Despite a clear need, it is estimated that a majority of health profession curricula have no IPE program in place [11]. Challenges to implementation of IPE such as alignment of learning activities, outcomes and training schedules contribute to the limited availability of IPE activities for students in health professions education across the globe [4,8,9,11,12,13,14,15]. University hospitals and other workplaces, where professionals and students work and learn from each other, offer a unique learning environment for IPE. However, implementation in a workplace setting seems particularly difficult because of the limits posed by existing workflow and organizational cultures [12,15,16,17]. For this study, we investigated the implementation of an IPE activity in a workplace setting, aiming to define design principles to support educators in adopting an evidence-informed practice and multiply IPE initiatives.

Although a workplace setting offers ample opportunities for IPE, several studies describe difficulties surrounding its implementation [1,4,12,13,14,15,16,17,18]. For example, each professional curriculum has its own program and aligning educational activities and learning objectives can be an organizational nightmare. Moreover, IPE implementation in a workplace setting often happens “on the run,” i.e. a new learning activity needs to be developed and implemented into an existing workplace with an established workflow, practice and culture. Often, this workplace is already under stress and professionals in the workplace may regard implementation as an extra burden and a possible threat to patient safety. Implementing IPE requires a readiness for change amongst students and staff. This includes the need for faculty members to embrace and nurture their collaborative self and act as positive role models [17,19]. Finally, the IPE activity needs to be well designed, i.e., guided by relevant educational theory, with well-defined learning goals and assessment methods [18,20,21,22]. To deal with this tension between IPE and the workplace routine, we believe IPE implementation should mature through a systematic, theory-driven, and reflective process.

Therefore, we used an Educational Design Research (EDR) approach to develop, implement and assess a theory-informed, context-sensitive IPE activity at a university hospital in the Netherlands [23,24,25,26]. Our aim was to implement a theory-driven, contextualized, clinical IPE activity, while elaborating design principles to support future implementation efforts in other workplace settings. Anticipating the challenges posed by the “on the run” implementation, we involved diverse stakeholders in an iterative process to co-construct this IPE activity. The structured reflection and analysis of the implementation process allowed for the description of practical design principles for successful implementation of an IPE activity within a workplace setting.

## Methods

### Design

**Figure 1:**
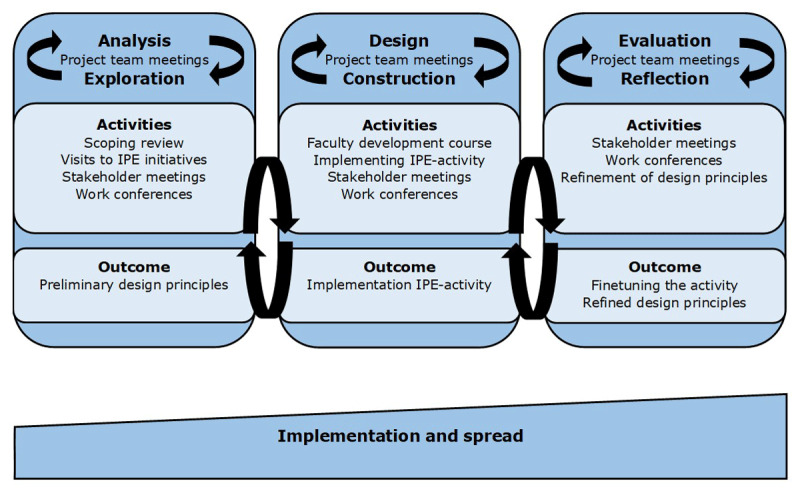
An educational design research approach to implementation of an IPE activity in a workplace setting.

We used an educational design research approach (EDR) to implement an IPE activity using an existing workplace setting as a starting point (Figure 1). EDR combines research with educational development to address complex educational problems improving our understanding of these problems as well as further enriching educational theory [23,24,25,26]. EDR consists of 3 phases: analysis/exploration, design/construction and evaluation/reflection, that are completed in an iterative, cyclic process gradually translating an idea into practice. Below we describe the general approach to the project, data collection and analysis. This is followed by a detailed description of each EDR-phase.

#### Setting, participants, and project team

The University Medical Center Groningen (UMCG) is a tertiary care teaching hospital in the Netherlands. This hospital had recently expressed the ambition to implement IPE, which contributed to institutional momentum for educational innovation. Two workplace educators, experienced with educational development from the department of obstetrics and gynecology were appointed as a project developer (HH) and a project leader (MV). Both received 0,1 full-time equivalent to work on this project. An existing workplace setting within this department was used as a starting point for implementation. HH and MV assembled a project team consisting of educators and researchers with different professional backgrounds to guide, coordinate and investigate the implementation process. Participation in the project team was open. However, we purposefully approached educators with different levels of experience and professional backgrounds, and from different training institutions in order to create a team of diverse professionals that was well embedded in the different curricula. The team consisted of a nurse (HH), midwives (RvdS, MG, AT), a gynecologist (MV) a resident in gynecology (LZ), as well as a research student (SP) and an experienced researcher in medical education (McdF). The project team scheduled regular meetings at 3–7 week intervals depending on the progress of the project. During the first meetings, the project team formulated a workplan for the project, describing the aim of the project, theoretical grounding and how the EDR-approach would be operationalized. The objective of the project was: “to implement an IPE activity where students from nursing, midwifery and medicine learn with, from, and about each other.” Aiming to take advantage of real clinical experiences to create learning opportunities, the project was grounded on Experience Based Learning theory [27].

#### Data collection and processing

Following the principles of the EDR, we engaged with theory and practice in an iterative process by employing a variety of activities. For the theoretical grounding of the study, we performed a scoping review that was regularly updated. To engage with practice, we visited examples of successful IPE initiatives at other institutions and involved stakeholders in the implementation process. We considered professionals in the workplace pivotal to the project and organized work conferences to involve them as described below. The project team documented observations and outcomes of these activities in notes, reports and reflective logbooks. In addition, HH kept a logbook, noting ideas and informal conversations relevant to the project. These documents formed a dataset that supported a meaning-making process during the project team meetings where we iteratively combined and analyzed observations and outcomes to formulate the next step in the implementation process. Reports from these meetings were added to the dataset for later analysis of the implementation process. For the first EDR-phase (exploration/analysis), this iterative process resulted in preliminary design principles for implementation. In the second EDR phase (design/construction) this process resulted in implementation of an IPE activity. In the third EDR phase (reflection/evaluation), this process served to finetune the activity and reflect on the implementation process. The project team re-examined the dataset to refine the design principles and reflect on the implementation process as described in the results. In the section below we elaborate on the activities employed, further specifying data collection and analysis for each activity.

### EDR-phase 1, Exploration and Analysis

#### Scoping review

We performed a scoping review to gain a theoretical understanding of IPE and its implementation. MV and HH performed a search in Pubmed and EBSCOhost using the search terms “implement* AND IPE”. Titles and abstracts (tiab) were screened, selecting studies published in peer reviewed journals in the English language between 2000 and 2022 that described implementation of IPE. Full text publications were retrieved and analyzed to identify recommendations for implementation of IPE in a workplace setting. Findings were discussed with the project team. During the project, the search was regularly updated by MV. The original search resulted in 454 articles of which 37 full text articles remained after tiab screening. Updating the search and including relevant references resulted in a total of 46 articles. Out of these 46 articles, 23 articles provided recommendations that could support the implementation of an IPE activity.

#### Visits to a successful IPE initiative

A previous study by Visser *et al* investigated clinical reasoning on an existing interprofessional obstetric ward [28]. This was considered to be a successful example of an IPE initiative because it has been in place for over seven years and has been part of several research studies. Two visits of one day to this ward were made by members of the project group (MG, RvS, HH). The aim of the first visit was to experience what an IPE activity could look like and to learn about the process of implementation. The second visit focused on the implementation process. The visits were structured around observing the daily routine on the IPE ward, including teaching and patient care; the observation was followed by conversations with educators in the workplace and students to address questions about the activity itself and its implementation, elaborating on issues raised by the scoping review and work conferences (described below). Examples of issues raised included how students were prepared for their placement, how they were supervised, how faculty was trained, as well as more technical issues such as access to electronic patient files and where students were seated in the ward. The members of the project team that visited this ward made reports and shared their experiences during the project team meetings.

#### Stakeholder involvement

We used the EDR-approach to connect the theoretical understanding of IPE with the experience of stakeholders, existing workflow and organizational culture. In order to coordinate which stakeholder should be involved at what point, the project team classified the stakeholders into three groups. The first group consisted of stakeholders with direct involvement, with high levels of interest and influence. This group consisted of healthcare professionals working on the ward and the ward manager. Because of their high level of interest and influence, the project team purposefully involved them in regular work conferences as described below.

The second group consisted of stakeholders with indirect involvement, with a lower level of interest and/or influence, who engaged with the process only when needed/invited. These were patients, students, other professionals within the department, and organizational managers. We aimed to involve these stakeholders in the implementation process to safeguard and align with their interests. We organized three-five meetings with different types of stakeholders throughout the implementation process to share findings, informing them about the development of the activity and asking for their input and suggestions. They were also encouraged to provide suggestions they deemed useful. The third group consisted of stakeholders with a strategic position in the hospital or in the healthcare region, such as leaders in healthcare and medical education. The approach to this group was similar to the second group, but meetings were less frequent (1–2 times). HH and MV processed the input and suggestions in a stakeholder analysis, describing conditions and requirements for developing and implementing the IPE activity. The stakeholder meetings created momentum and urgency within the organization to change, while devising an actionable plan.

#### Work conferences

Over a period of 10 months, the project team organized eight work conferences with the first group of stakeholders (i.e. professionals from the workplace). The aim of these conferences was to involve professionals from the workplace in the implementation process by sharing information about IPE and the implementation process, simultaneously obtaining their input on the process. Each conference started with an update on the progress of the project, initially explaining the concept of IPE and describing the steps taken so far. Next, an interactive and democratic dialogue explored two topics: IPE conceptualization and implementation in the workplace. This dialogue served to obtain input from these stakeholders, making use of their experience in the workplace. The project team took advantage of scheduled, continuous professional development sessions to organize the work conferences. Each work conference was attended by the project team and between 20–25 professionals from the workplace at a time. To facilitate maximal attendance during the covid-19 pandemic, conferences were hybrid, using mentimeter to facilitate interaction and documentation. The project team prepared and guided the work conferences. In addition, the project team documented proceedings of the conferences by noting observations and impressions during the conference. Presentations from the work conferences and the documented proceedings from the conferences were stored to support analysis during meetings with the project team in each EDR-phase.

The work conferences played an important role in the implementation process. First, the conferences created a safe space for stakeholders to learn about, from, and with each other while reflecting on how to enact change. This democratic dialogue culminated in mutual empowerment, where professionals from the workplace felt open to share concerns on patient safety, workload and bridging the gap between professional cultures. It also facilitated a constructive discussion on how these concerns could be addressed in the construction and design of an activity. In addition, the different cultural layers became an explicit part of project team discussions, work conferences and the IPE supervisor course (described below). Cultural differences were celebrated, valued as assets in the implementation process. These effects of the work conference created additional momentum for change.

### EDR-phase 2 Construction and Design

#### Faculty Development

In response to an identified need during the work conferences, the project team developed a course to support IPE enthusiasts to act as supervisors and role models in the IPE unit. The aim was to empower faculty members to seize opportunities for nurturing the growth of a collaborative attitude in students by providing interprofessional feedback based on cultural humility, i.e. awareness of diversity with an active attitude permeated by self-criticism, which challenges pre-conceived assumptions and is curious about others’ perspectives. The course consisted of three modules of four hours, covering the development of educational skills and the fundaments of interprofessional education and collaboration. As some faculty had limited experience in teaching, modules 1 and 2 focused on general aspects of faculty development such as adult learning, teaching in small groups, providing feedback etcetera. Modules 1 and 2 also discuss how these general aspects link to IPE. The third module focused on IPE specifically, addressing learning objectives, assessment, cross professional feedback and role modeling. Appendix 1 provides a description of the course. At this stage of implementation, the project team decided first to train the early adopters amongst workplace professionals. Participants who finished the course were awarded the title of *IPE supervisor*, recognizable by colleagues and students. Reflective feedback sessions during the course provided valuable input for the design of the IPE activity, especially on how IPE competencies described in the literature could be translated to the workplace.

#### Implementation of the IPE activity

Informed by the preliminary design principles resulting from EDR phase 1, we constructed and designed an IPE activity. This was again an iterative process, in conversation with the workplace that complemented the design principles. This conversation allowed us to address concerns expressed by the workplace, respond to a perceived need for faculty development, and address logistical issues such as alignment of schedules and arranging workstations for students. The activity was introduced in three steps covering EDR-phase 2 and 3, gradually phasing the activity into the existing workflow and allowing refinement of the activity in the process. The first step was introduction of the activity for a period of two weeks. To minimize the impact of its introduction on the workplace, this step occurred after the activity had matured through several iterations in EDR-phase 1&2. After these two weeks the activity was stopped and evaluated. During stakeholder meetings and work conferences, we evaluated the impact of the activity on patient care, workflow and student learning. Findings from the evaluation were documented and discussed with the project team, informing refinement of the activity. The evaluation resulted in a few logistical changes, improving the access of the students in midwifery and medicine to electronic patient files. In the second step, the IPE activity was implemented in the workplace and evaluated after six weeks, as part of the 3^rd^ EDR-phase of evaluation/reflection.

### EDR-phase 3, reflection and evaluation

#### Evaluation of the IPE activity

Approximately six weeks after implementation we evaluated the impact of the activity on patient care, workflow, and student learning during stakeholder meetings and work conferences. The approach, including data collection and processing was similar to step 1 of the implementation process. Although we made no further adaptations to the activity based on this evaluation, this evaluation resulted in a decision to provide faculty development to all professionals in the workplace, allowing more flexible planning in the future. After this, the implementation was considered to be final and in step 3 of the implementation process, ongoing evaluation became part of a regular process of quality control. The resulting IPE activity is described in Appendix 2.

#### Refinement of design principles

Data collection and analysis occurred alongside the implementation process and thereafter in meetings with the project team, resulting in a large dataset consisting of documentation from the activities, reports from the project team meetings and a logbook containing ideas and informal conversations relevant to the project. After implementation of the IPE activity was finalized, the project team continued with scheduled meetings to review the implementation process. In these meetings, the project team re-examined the dataset, discussing key features to support implementation in the workplace and refining the design principles. As a first step in refining the design principles, HH, MV and LS listed the preliminary design principles, including a description of how this design principle had been applied during the implementation. Next, each team member reflected on each principle and related description individually. Using surveymonkey, an online survey tool, team members could agree or disagree with the design principle, and could make a comment, question or suggestion to improve it. This resulted in an iterative process, where the project team further refined design principles and described the underlying mechanism explaining how a design principle could facilitate IPE implementation. This step of individual reflection and plenary discussion was repeated until consensus was reached on all the principles after three meetings. Aiming to support application of these design principles in practice, we also expanded on the concerns expressed by participants of the work conferences and how they could be addressed.

### Research team and reflexivity

The research team consisted of members with different backgrounds and perspectives that complemented each other during the study. The team included a gynecologist (MV), a resident in gynecology (LZ), midwives (RvdS, AT, MG), and a nurse (HH), all working in HPE. This allowed for different professional perspectives to be taken into account in the EDR process. SP is a medical student with an interest in medical education and research who provided input during the study from a student perspective. MdCV is an internal medicine specialist and professor in medical education, experienced in qualitative research. MdCV facilitated the research process by, identifying relevant data, contributing to analysis and critically revising the findings.

### Ethical considerations

For this study, ethical approval was obtained by the ethical review board Dutch society for medical education (NVMO-ERB, file number: 2023.1.3).

## Results

In the first EDR phase (analysis/exploration), activities such as the scoping reviews, visits, and work conferences allowed the interprofessional project team and stakeholders to develop a shared understanding of what an IPE activity could look like and how it could be implemented. The project team deemed development of a shared understanding pivotal to the process and accepted the time and iterations needed for the process to develop. Nurturing enthusiasm and trusting this process allowed stakeholders, and professionals in the workplace in particular, to gradually engage with the project. As this process continued, stakeholders became more and more involved and empowered to contribute to the development of the activity. They started to share their concerns as well as their ideas on how to address these concerns. Analysis in the first EDR-phase (analysis/exploration), resulted in 12 preliminary design principles that informed the next EDR-phase (design/construction) where an IPE activity was implemented in the workplace.

The third EDR-phase (evaluation/reflection) served to finetune the IPE activity and to refine design principles. Table 1 describes 14 refined design principles to support context sensitive IPE implementation other settings. To support application of these design principles in practice, we expanded on the concerns expressed by stakeholders identifying three overarching concerns relating to implementation as a change process: guaranteeing patient safety, aligning with the workflow in the workplace, and bridging the gap between professional cultures.

**Table 1:** Refined design principles for implementation of an IPE activity in a workplace setting.


1.	Set a feasible, clear, and meaningful objective to articulate the need for change and communicate to stakeholders.

2.	Make the project evidence informed and theory driven to align the project’s goals with the needs of the stakeholders.

3.	Stimulate transformative participation of stakeholders from all professions and backgrounds to facilitate ownership, enact change and contribute to professional development.

4.	Be aware of the several cultural layers intersecting in IPE activities (local, professional, and organizational cultures).

5.	Support, engage and develop faculty members who have the ability to break silos, act as role models, and are able to create trust and respect in a safe learning environment.

6.	Align learning outcomes with available IPE competency frameworks as well as the context of the workplace.

7.	Design assessment methods incorporating formative and reflective feedback to support the development of the complex competencies related to IPC.

8.	Position an IPEactivity within an authentic context, i.e., as close as possible to the reality of the practice.

9.	Facilitate and stimulate informal interaction between students.

10.	Balance guaranteeing patients’ safety with attributing responsibility to students.

11.	Evaluate the implementation, switching gears between the phases of EDR: analysis/exploration, design/construction and evaluation/reflection.

12.	Align the educational activity with the workplace workflow and routine and seize opportunities to improve IPC in the process.

13.	Nurture a growth mindset – obstacles are opportunities to understand, clarify and improve the intervention.

14.	Stay positive, nurture your enthusiasm, and trust the process.


### Implementation as a change process

We identified three overarching concerns related to the realization that IPE implementation functioned as a change process: patient safety, workflow and culture. For healthcare professionals that are deeply involved in patient care, educational innovation may be perceived as a threat to patient safety and an extra burden to an already overwhelming workload. These concerns were mainly expressed during the work conferences. In the first EDR-phase (analysis/exploration), participants expressed concerns such as: “supervision of students may cost a lot of time” and “students may warn us too late if a problem arises.” In the second EDR-phase (design/construction) participants started to provide constructive input on how these concerns could be addressed, such as: “reorganizing rounds so that it becomes more effective” and “getting acquainted to reduce the barrier to ask for help”. The iterative nature of the EDR-approach, contributed to a dialogue with stakeholders that resonated with the design principles and helped to address these concerns.

#### Change and patient safety

Stakeholders, especially the healthcare professionals working on the ward, acknowledged that students are in the process of becoming professionals. They agreed that students need space to act autonomously, with adequate supervision to guarantee patient safety. However, stakeholders expressed concern that the increased number of persons (students and professionals) on the ward would create a demand for extra supervision, which could distract from patient care and result in “fragmentation of care, posing a risk to patient safety”. In the first EDR-phase, we identified the need to *balance patients’ safety with attributing responsibility*. Finding this balance was supported by the EDR-approach. For instance, during the work conferences, we constructed a shared understanding of how student participation could impact patient care [3,29]. In the second EDR-phase, this shared understanding allowed for a conversation about how patient safety could be balanced with student participation and resulted in a description of the requirements to guarantee patient safety such as a structure for supervision. This conversation on patient safety and student participation was further supported by other design principles (principles 1, 3, 9, 12, 13 and 14 – Table 1). The engagement of diverse stakeholders in the conversation about patient safety in the new IPE unit resonated with the idea of participation as empowerment, a characteristic of *transformative participation, i.e*. when stakeholders contribute to collective decision making, planning and action (principle 3 – Table 1) [30].

#### Change and workflow

Another concern was how the implementation of an IPE activity would impact the workflow and clinical routine. For instance, the workload was a recurrent topic in the work conferences. In the work conferences in the first EDR phase, the project team and participants created a shared understanding that the implementation of an IPE activity could result in work being *different* but not *more*. This understanding reassured participants and positively contributed to their engagement, opening a conversation during the first and second EDR phases on how an IPE activity could be aligned with the workplace and workflow. Interestingly, the conversation revealed some vulnerabilities of the existing workflow that could be seized as opportunities to improve interprofessional collaboration (IPC). For example, patient rounds were not scheduled and happened randomly according to the availability of different professionals. Stakeholders perceived this as inefficient and undesirable. Earlier attempts to schedule a set meeting with the participation of all professionals to discuss all patients had failed. Using the implementation project as a driver, a new structure for patient rounds, with a set time planning was implemented before the implementation of the IPE activity, improving the efficacy of patient rounds and IPC on the ward. The improvement of the workflow secondary to IPE implementation was strategic to improve stakeholders acceptance of change.

#### Change and culture

The cultural differences between healthcare professions was a recurrent topic throughout the implementation process. The different professions in the workplace and elsewhere in the organization have different values and attitudes. In addition, values and attitudes differ between students and professionals. These differences created an intersectionality of interests and concerns that needed to be acknowledged in order to facilitate the change process required for IPE implementation. Throughout each EDR phase, the work conferences served to explore these differences, acknowledging their relevance to the implementation and creating a space to discuss possible means to bridge cultural differences. Accommodating these differences sometimes required the project team to take a step back in the process, and to switch gears between different phases of EDR, rather than maintain the cyclical approach that is characteristic of EDR. This active approach to cultural differences allowed the project team and stakeholders to challenge pre-conceived assumptions and to find common ground, while respecting different perspectives. Besides *awareness of the several cultural layers intersecting IPE*, several other design principles (principles 3, 5, 10, 13 and 14 – Table 1) contributed to a setting of cultural humility that supported the change process.

## Discussion

We investigated implementation of an IPE activity in a workplace setting using an EDR approach and defined 14 design principles that can support the implementation of IPE *in* and *with* the workplace. Illustrating their potential utilization and further adding to the current understanding of IPE implementation in a workplace setting, we also identify three overarching concerns related to the implementation process and describe how we addressed these concerns. The EDR approach used for this study allowed us to create a shared understanding with stakeholders in the workplace, resulting in a meaningful dialogue that resonates with the concept of transformative participation of stakeholders [30]. In this discussion, we reflect on our findings by addressing IPE implementation as an evidence informed and evidence informing process.

### Implementing IPE as an evidence informed and evidence informing process

The implementation process in this study illustrates that evidence informed development of education in a workplace setting benefits from a mutually reinforcing dialogue between what we know about IPE and how IPC is shaped in practice. Taking the time in the first EDR phase to perform a scoping review, visit other IPE initiatives, and engage with stakeholders allowed the team to construct a shared theoretical understanding of IPE, which prevented the repetition of previous mistakes and sharpened the implementation process. This dialogue also facilitated the co-creation of a common academic “language” to name the needs of the different healthcare professionals involved in the process. This collaborative atmosphere made the different healthcare professionals comfortable to keep their professional identities while reflecting on the vital elements necessary to developing an interprofessional identity, i.e. “a social identity that is shared by members of different professions belonging to the same team” [31]. The prolific discussions that followed set the tone for co-designing and co-creating the IPE activity.

This evidence informed process became evidence-informing by the active participation of the team members and their reflections throughout the implementation process and after. This theoretical endeavor had practical implications. For example, the project team noticed that a conversation with the different stakeholders required flexibility in the implementation process and decided to invite professionals from the workplace to take leading positions in the process. Besides, although EDR is often described as a circular process, the project team perceived the process more as *switching gears between the different phases of EDR* depending on the needs of the project team and stakeholders. In addition, the project team learned to *nurture a growth mindset* and to be open for different ideas and suggestions from different stakeholders, even when the latter were initially resistant to change. Nurturing enthusiasm and trusting the process paved the way for a collaborative search for shared ground, which further empowered stakeholders to participate and contribute. We learned how an existing workplace with an established workflow and culture might be the departure point to transform theory into motion and ideas into transformative actions, promoting in-depth understanding of the challenges related to IPE implementation.

### Implementation facilitated by transformative participation

IPE can be implemented top-down, when organizational leaders take initiative for and control over the implementation process [1,12,15]. However, implementing IPE top down, prescribing to professionals what and how to teach, can lead to passive and less engaged faculty who may end up opposing change [32]. Conversely, empowering workplace professionals to participate and influence the design and implementation process provides valuable input and fuels further engagement creating a platform for change [33].

Sarah White describes four levels of participation; nominal, instrumental, representative and transformative [30]. Nominal participation is where stakeholders are informed about but have no influence over a process. Nominal participation is often associated with tokenism and is used to legitimize decisions. With instrumental participation, stakeholders’ efforts are required for a project to succeed, but they are not involved in the process. With representative participation, stakeholders have a voice, but do not have a role in decision making. Transformative participation is more challenging and requires empowerment of all stakeholders to have a shared and democratic control over the process. Transformative participation implies that stakeholders are empowered to participate and contribute to design and decision making, resulting in a cocreation process [30].

During the IPE implementation, the project team learned how the empowerment of people from the workplace could support the development of the activity and facilitate a change process. Especially during the first EDR phase, the project team took time to iterate between analysis and exploration, which contributed to transformative participation. Trusting the process, allowing time to discover the possibilities for- and challenges to IPE implementation contributed to stakeholder empowerment. Their involvement in construction/design of the activity may also explain why the implementation required few adaptations of the activity. Equipped with knowledge, a common “language” and purpose, the team engaged in an EDR protocol to implement an IPE activity *with* the “workplace” and not *for* or *despite* the workplace.

#### Transformative participation and EDR

In our experience, the EDR approach is an effective research tool with the additional benefit of accommodating participation because it requires a conversation with the people in the workplace. In this study, the work conferences proved to be a place where this conversation could shape an implementation process that was defined and supported by stakeholders. Implementation of IPE in different contexts may not require work conferences as described here but will benefit from an approach that allows stakeholders to contribute to collective decision making and action in a process of transformative participation.

#### Transformative participation and IPE

There is a resemblance between transformative participation and IPE, where one mirrors the other, that can be used to further improve IPE. IPE aims to develop the competencies required for IPC that empowers different professions to contribute to and have control over the care process in a collaborative effort that values complementary expertise. Both concepts require far-reaching collaboration where different groups rely on other groups in different power positions with different access to decision making [5,30]. In patient care for example, the healthcare team not only relies on a nurse to operationalize a care plan, but also for the nurse to contribute to the design of that care plan. This requires other members of the healthcare team, such as doctors, to relinquish control and power and is often perceived as difficult [34]. As with transformative participation, health profession education is struggling to overcome hierarchy and power dynamics [5,35,36,37,38]. In a way, IPC requires transformative participation where healthcare professionals are empowered to address workplace systems and structures [5]. In this study, the EDR approach supported transformative participation, allowing different professions to shape the implementation process [13]. We believe that this collaboration at the educational level can be inspirational to improve the collaboration at the clinical level. The impact of the implementation process on IPC is currently being investigated and could shed light on how transformative participation and a shared language could be used to address hierarchy and power structures in IPE and IPC.

### Next steps

This study investigated the implementation process of an IPE activity that resulted in an IPE unit where students of midwifery, nursing and medicine care for patients together. As a next step of this EDR project, studies have been initiated to systematically investigate the impact of the activity on participants, professionals, patients and the workplace as a learning environment. Using mixed methods approaches with specific and qualitative measures these studies investigate the development of interprofessional competencies in students as well as the development of professional and interprofessional identity and critical thinking skills. Focusing on professionals we are investigating the impact of the IPE activity on IPC and job satisfaction. Studies investigating patient experience with IPE and their perspective on interprofessional learning have recently been initiated.

### Implications for practice and future research

Implementation of IPE is a challenging change process and investigating implementation initiatives is difficult as the context of different workplace setting complicates the formulation of generalizable design principles. To our knowledge, we are the first to use an EDR approach to implement a theory driven, context sensitive IPE activity in a workplace setting allowing us to connect theory with practice. Reflecting on this process we describe 14 generalizable design principles. In addition, we explored the concerns of professionals in the workplace that can hinder implementation and describe how these concerns can be addressed. The iterative aspect of EDR proved to be valuable in this as it facilitated a conversation with the workplace, resulting in transformative participation of professionals in the workplace in co-creation of an activity that is aligned with the workplace. When future initiatives use an approach such as EDR they can address complex educational problems, improve understanding of these problems and enrich educational theory as well as benefit from using an approach that facilitates change.

Many of the design principles described are related to different values and beliefs that can enable or limit implementation and provide directions for further research. For example, the importance of power dynamics in healthcare education and collaboration is widely recognized as a topic for further research [5,35,36,37,38,39]. Besides power dynamics, insights into the different professional cultures and how differences impact collaboration may support the design of educational practices to bridge students these differences, turning them into professionals capable to navigate their landscape of practice [2,40,41].

### Strengths and limitations

A strength of this study is the EDR approach to implementation of an IPE activity that allowed for a reciprocal evidence informed, evidence informing implementation process shaped by the workplace [23,24,25,26].

An important limitation is the setting for this study on a maternity ward in a tertiary hospital in a high- income country. Although the design principles described here do not focus on aspects specific to this setting, it is possible that other contexts might bring specific challenges not accommodated by our design principles. Possibly, a study on a different ward, in a different setting could lead to the tightening up of the definition of the principles or to the addition of new ones.

## Conclusion

This study describes 14 generalizable design principles to support implementation of IPE activities in workplace settings. The design principles support an evidence informed approach to implementation that considers educational aspects relating to learning outcomes, assessment methods, faculty development and facilitates informal interaction between students in a workplace setting. In addition, we describe three overarching concerns supporting a change process accommodating patient safety, workload and culture. Using an EDR approach, operationalized by the appointment of an interprofessional project team and the organization of work conferences, enabled us develop a shared language and facilitated transformative participation of stakeholders that became part of the development process. Using a similar approach in future implementation projects can help to refine these design principles, shedding light on the ways IPE can contribute to improving patient care in different contexts.

## Data Accessibility Statement

The data is available upon request. The data contain an extensive documentation of the implementation process, including a logbook and stakeholder analysis. These documents contain names of stakeholders and we did not ask for permission to share these documents in an open repository.

## Additional Files

The additional files for this article can be found as follows:

10.5334/pme.1546.s1 Supplemental material 1. Preliminary Design Principles.

10.5334/pme.1546.s2 Supplemental material 2. Learning outcomes of the IPE activity.

10.5334/pme.1546.s3 Supplemental material 3. Example of a day schedule.

10.5334/pme.1546.s4 Supplemental material 4. Boxes 1 and 2.
