# Supplementary material for: Implementing IPE in a Workplace Setting: Educational Design Research Promotes Transformative Participation
Source: Perspect Med Educ. 2025 Jan 23;14(1):31–43. doi: 10.5334/pme.1546 (PMC11758813; doi:10.5334/pme.1546)
Supplement: Supplemental material 1. — Preliminary Design Principles. [file pme-14-1-1546-s1.pdf]

Table 1<br>**Preliminary design principle**
1. Set an objective and articulate reason for change (pfadenhauer 2017; WHO 2010; mcCleod 2015; reeves 2016; kali 2020)
2. Use an evidence informed approach (Clark 2009; mcCleod 2015; WHO 2010; reeves 2017; Kali 2020)
3. Inform development on educational theories (Clark 2009; McCleod 2015; bogossian 2022; reeves 2016, haen 2015)
4. Involve stakeholders from all professions and backgrounds (Diggele 2020; bogossian 2022; pfadenhauer 2017; reeves 2016)
5. Develop faculty that can act as a role model, that are skilled as a teacher, facilitating trust and respect. (Bogossian 2022; McCleod 2015, Diggele 2020, Visser 2017; maddock 2022, Hofmeyer 2022; Hamoen 2020; reeves 2016)
6. Constructively align learning outcomes with IPEC competencies (WHO 2010; Reeves 2017; IPEC 2016, CIHN 2011; McCleod 2015, Diggele 2020; visser 2017; maddock 2022; Hofmeyer 2018, Clark 2009; bogossian 2022; pfadenhauer 2017)
7. Align content of an IPE activity with learning principles of adult learning and interprofessional learning. (McCleod 2015, Diggele 2020; visser 2017; maddock 2022; Hofmeyer 2018, Clark 2009; bogossian 2022)
8. Position an IPE activity within an authentic context ((McCleod 2015, Diggele 2020; visser 2017, Hofmeyer 2018, dornan 2019)
9. Create a safe learning environment (McCleod 2015, Diggele 2020; reeves 2016)
10. Address culture and organization (Visser 2017; Hamoen 2020)
11. Develop an assessment method (McCleod 2015, Diggele 2020; visser 2017; clark 2009)
12. Evaluate the implementation (Kinsey 2020, Diggele 2020; McCleod 2015) (as part of EDR)
