# Supplementary material for: Implementing IPE in a Workplace Setting: Educational Design Research Promotes Transformative Participation
Source: Perspect Med Educ. 2025 Jan 23;14(1):31–43. doi: 10.5334/pme.1546 (PMC11758813; doi:10.5334/pme.1546)
Supplement: Supplemental material 2. — Learning outcomes of the IPE activity. [file pme-14-1-1546-s2.pdf]

Implementing IPE in a workplace setting. Supplemental Material 3, learning outcomes

Basic learning outcomes

All students participating in the IPE-unit are required to formulate learning objectives based on 6 predefined basic outcomes. Students who are doing well have the opportunity to extend on this and formulate objectives based on 6 advanced learning outcomes.

Basic outcome 1 Communication: The student has an open communication style, with respect for the other students and develops a constructive partnership

Basic outcome 2 Identity/Role: The student recognizes his own role and the role of the fellow student from other programs within the IPE unit

Basic outcome 3 Collaboration: The student can collaborate with fellow students in patient care and enable effective patient outcomes

Basic outcome 4 Values/norms for Interprofessional Working: The student recognizes the expressed needs of all parties within the IPE student group and the care recipients and can reproduce them.

Basic outcome 5 Professionalism/Leadership/Reflection: The student visibly reflects on their own functioning within the unit and gives feedback to fellow students. Students can assess each other and use forms that are offered within their own study programme.

Basic outcome 6 Professionalism/Leadership/Reflection: The student acts as chairman during the interviews and visits, ensures that the administration is kept efficiently/adequately and communicated in a timely manner.

Advanced learning outcomes

Advanced outcome 1 Communication: The student can communicate in a reactive and responsible way with the patient and family members

Advanced outcome 2 Identity/Role: The student always exhibits 'professional behavior' aimed at improving interprofessional cooperation and takes initiative in this

Advanced outcome 3 Collaboration: The student can properly consult other healthcare professionals for their skills and knowledge

Advanced outcome 4 Professionalism/Leadership/Reflection: The student can reflect on her own performance and actively seeks input from fellow students, professionals, patients and their family members

Advanced outcome 5 Professionalism/Leadership/Reflection: The student looks for the best available substantiation for action and takes a critical and responsible attitude

Advanced outcome 6 Professionalism/Leadership/Reflection: The student knows the importance of health promotion and can transfer this knowledge and motivation to patient and fellow students
