# Supplementary material for: Implementing IPE in a Workplace Setting: Educational Design Research Promotes Transformative Participation
Source: Perspect Med Educ. 2025 Jan 23;14(1):31–43. doi: 10.5334/pme.1546 (PMC11758813; doi:10.5334/pme.1546)
Supplement: Supplemental material 3. — Example of a day schedule. [file pme-14-1-1546-s3.pdf]

Implementing IPE in a workplace setting. Supplemental Material 3.

Day schedule for students (midwifery and medicine) on the IPE-unit

7.30 Meet up at the ward and check which patients have been assigned to whom. Attend the handover meeting at 7:45. Get an overview of the patients on the ward and identify learning opportunities.

8:30 prepare patient rounds together with nursing students. Aim for a conversation where you can share ambiguities and challenges. Formulate remaining questions and a plan for the day, to be discussed with the attending midwife.

9:00 Maternity patient rounds on paper. The nursing students provides an overview of your patients according to the SBARR structure. You can ask for clarification where needed. During the R (Repeat) of the SBARR you discuss the tasks and task distribution for today.

9:00 Pediatrician patient rounds on paper. Newborns with involvement of the pediatrician are discussed is using the same approach as for maternity patient rounds.

9:30 Write notes in the electronic patient file. Discuss and finalize with IPE supervisor

11.30 Physical patient rounds, Providing information to patients in an interactive conversation, support mother and newborn. Draft discharge letter if needed.

12.15 Lunch break together with fellow students

12.45 Updating administration, call primary care givers (midwives) in case of a discharge for a verbal handover, prepare patient rounds for tomorrow

14:00 Evaluate with fellow students if all tasks have been completed, collaborate on any remaining tasks or plan to transfer tasks to the late shift.

14:45 Room for further discussing with patients, addressing any questions or discharge. Evaluate the day with supervisor.

15:00 Patient handover to the midwife of the late shift.

15.20 Transfer to the evening shift by the nursing student

15.30 Short teaching moment for an by students.

16.00 End of day meeting
