# Supplementary material for: Implementing IPE in a Workplace Setting: Educational Design Research Promotes Transformative Participation
Source: Perspect Med Educ. 2025 Jan 23;14(1):31–43. doi: 10.5334/pme.1546 (PMC11758813; doi:10.5334/pme.1546)
Supplement: Supplemental material 4. — Boxes 1 and 2. [file pme-14-1-1546-s4.pdf]

Box 1. IPE supervisor course

The course consisted of 3 modules of 4 hours each. Sessions were interactive and reflective, using a workshop format. Preparational material consisted of youtube video's on specific topics, including video's on IPE made by the institution (Introduction to interprofessional collaboration and interprofessional education - YouTube). Each session ended with a reflective formative assessment.

Module 1 focused on group formation, the importance of IPE and an exploration of the profile of an IPE-supervisor. Reflective discussions explored challenges and solutions to HPE and IPE on a clinical ward. Learning objectives for this module focused on basic teaching competencies and an introduction on IPE. After the module, participants were able to:

- Explain principles of experience based learning and adult learning
- Explain the current understanding of principles underlying IPE and IPC
- Provide a short presentation using a set-dialogue-closure structure

Module 2 focused more on teaching in small groups and workplace learning as well as assessment and how to provide feedback. In a reflective discussion, participants further explored possible similarities and differences between teaching within a single profession, and IPE. Learning objectives for this module focused on basic teaching competencies and improved understanding of IPE. After the module, participants were able to:

- Teach in small groups
- Teach in a workplace setting
- Provide feedback using Pendleton rules
- Explain similarities and differences between IPE and single profession education

Module 3 focused on IPE in depth and explored aspect such as learning outcomes, related behavior, reflection and assessment. In reflective discussions participants explored cross-professional feedback and differences in professional culture between nurses, midwives and doctors and formulated an approach to bridge these differences. Learning outcomes for IPE were discussed including related observable behavior and resulted in co-creation of an approach to assessment on the ward. Learning objectives for this module focused on teaching IPE and the role of IPE-supervisors. After this module, participants were able to:

- Describe learning outcomes and assessment methods for IPE
- Describe their position as professional teacher and role model
- Describe differences between professional cultures

**Box 2.** Description of the IPE-activity
This study culminated in the implementation of an IPE-unit on a maternity ward of a maternity hospital. The ward specifically cares for women and newborns after birth. Approximately 4.000 women and newborns visit the ward each year with pathologies varying from mild to severe. In the IPE unit, 4 students collaborate and care for 4 patients. Students in nursing are paired with either a student in midwifery or medicine. Each pair takes care of 2 patients under supervision of an IPE supervisor. During their placement, students care for patients in the professional role of their training. Nursing students measure vitals, support mobilization, hygiene and breast feeding, and present their patient during the rounds. Students of midwifery and medicine check on patients, collect and interpret information during rounds. Together students formulate and execute a care plan, discuss the plan with patients and attend to administrative tasks. Each day there is an opportunity for informal interaction during lunch. During the day there are fixed moments for supervision but students can also approach a supervisor as required. At the beginning and end of the week supervision meetings are more extensive to discuss learning objectives and for reflective assessment respectively. The learning outcomes and an example of a day schedule are available as supplemental material 2 (Supplemental Material 2 and 3 respectively). Aligning with schedules of their training, two students in nursing attend the unit at a time for a period of 6-8 weeks. This allows them to get familiarized with their role as part of the care team. One student in midwifery and one in medicine attend the unit at a time, allowing them to get a basic understanding of patient care and their role as part of a care team.
